# Cyclophosphamide-Induced Lung Injury

**DOI:** 10.1016/j.ekir.2018.11.001

**Published:** 2018-11-10

**Authors:** Dan Pugh, Tariq E. Farrah, Peter J. Gallacher, David C. Kluth, Neeraj Dhaun

**Affiliations:** 1 University/British Heart Foundation Centre of Research Excellence, Centre of Cardiovascular Science, Queen's Medical Research Institute, University of Edinburgh, Edinburgh, Scotland; 2 Department of Renal Medicine, Royal Infirmary of Edinburgh, Edinburgh, Scotland

## Introduction

Antineutrophil cytoplasmic antibody (ANCA)−associated vasculitis is a multisystem autoimmune disease that often involves the lungs. It can be serious and sometimes fatal, and requires prompt recognition and treatment. Cyclophosphamide is a well-established alkylating agent that is widely used in the treatment of ANCA vasculitis. The most common side effects of cyclophosphamide are infection and the risk of malignancy.

## Case Presentation

**Figure 1:**
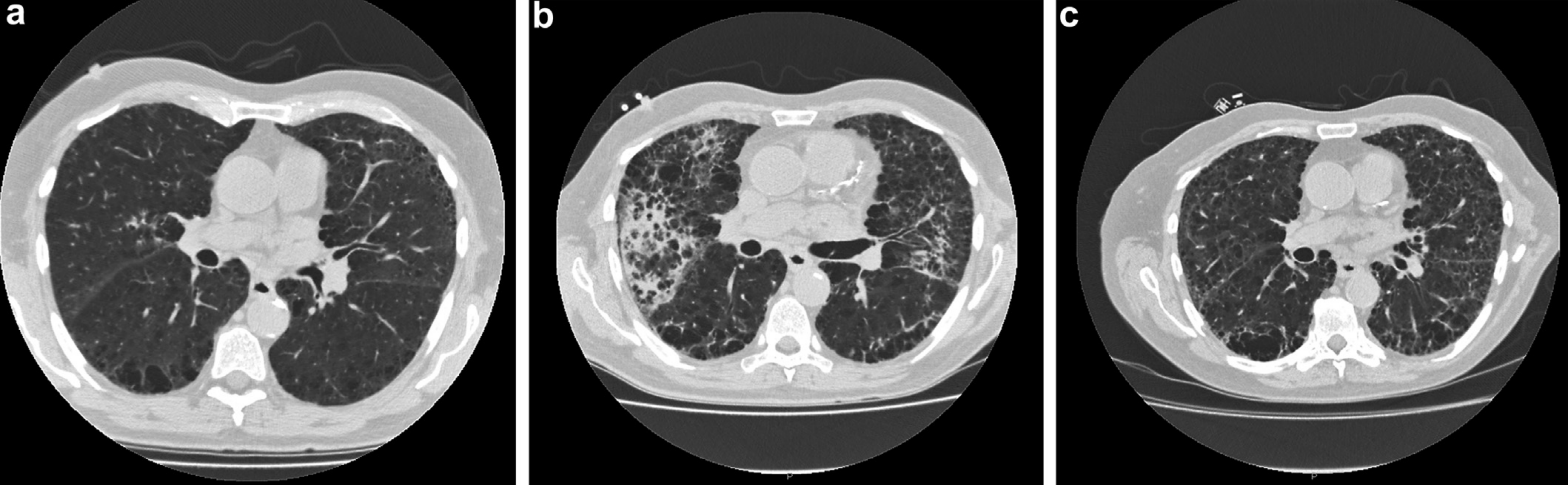
(a) Initial computed tomogram with mild changes, notably extensive emphysema, and bi-basal peripheral–ground-glass changes. (b) Computed tomogram from 4 months later (after initiation of cyclophosphamide), with severe centrilobar and paraseptal emphysema with new inflammatory changes. (c) Improvement in the changes seen in (b), 3 months after cyclophosphamide withdrawal.

A 61-year-old man, a lifelong smoker, was investigated in the respiratory outpatient clinic for progressive breathlessness. Pulmonary function tests at the time revealed a forced expiratory volume in 1 second (FEV_1_) of 2.84 L (predicted 3.2) and a forced vital capacity (FVC) of 4.9 L (predicted 5.1) with an FEV_1_/VC of 0.58. Based on these values, he was diagnosed with chronic obstructive pulmonary disease. His breathlessness continued to worsen and was associated with reduced exercise tolerance. As a result he underwent a high-resolution computed tomography (HRCT) scan of the lungs 2 months later. This showed extensive emphysema and bi-basal peripheral–ground-glass changes with possible honeycomb cyst formation (Figure 1a). Serum creatinine was normal at this time. The patient re-presented 2 months later with symptoms of lethargy, worsening breathlessness, and numbness affecting his left foot.

Physical examination revealed bi-basal fine expiratory crackles in the lungs alongside a mononeuritis multiplex. Renal function was severely impaired with a serum creatinine of 13.01 mg/dl (normal range 0.60–1.10) and C-reactive protein (CRP) was elevated at 93 mg/dl (normal range 0–5). Myeloperoxidase antineutrophil cytoplasmic antibody (ANCA) titers were raised at >100 IU/ml (normal range 0–5). The patient went on to have a renal biopsy. This showed an active segmental and necrotizing glomerulonephritis with evidence of significant tubular atrophy and interstitial fibrosis. Overall, the clinical diagnosis was of an ANCA-associated systemic vasculitis, most in keeping with microscopic polyangiitis.

Despite the significant chronic renal damage, the patient was treated with a combination of prednisone (1 mg/kg/d), plasmapheresis, and i.v. cyclophosphamide in addition to hemodialysis. His clinical condition improved significantly, in particular his shortness of breath. However, he remained dialysis dependent. He was discharged home with a plan to continue outpatient treatment with i.v. cyclophosphamide alongside a reduction of his prednisone dose. A repeat HRCT before discharge showed no new changes compared to the HRCT from a few months earlier.

The patient re-presented 6 weeks later with marked dyspnea. Despite 14.5 L of fluid removal on hemodialysis and treatment with broad-spectrum antibiotics, he remained dyspneic. Results of sputum and blood cultures as well as respiratory viral swabs were negative. Results of investigations for *Pneumocystis jirovecii* pneumonia were also negative. Diffuse alveolar hemorrhage was considered, although imaging was not consistent with this, hemoglobin was stable, and there was no externalization of blood. The patient was not fit to undergo bronchoscopy. An HRCT scan of the lungs was reported as showing severe centrilobar and paraseptal emphysema with super-added inflammatory changes (Figure 1b), significantly worse compared to a scan 2 months earlier. The patient had received 2 doses of i.v. cyclophosphamide, and it was believed that this was implicated. Following its withdrawal, the patient’s symptoms and exercise tolerance gradually improved. An HRCT 3 months later showed resolution of the lung changes seen on CT immediately before cyclophosphamide cessation (Figure 1c). The patient was diagnosed with cyclophosphamide-induced acute pneumonitis. His prednisone dose was increased transiently following withdrawal of cyclophosphamide.

## Discussion

Cyclophosphamide is an established alkylating agent that is widely used in the treatment of ANCA-associated vasculitis and in hematological malignancies as part of a chemotherapy regimen. Its most commonly recognized side effects are those of infection and the long-term risk of malignancy. Pulmonary side effects are rare (<1%) and are dose related.^1^ They manifest as either an early-onset pneumonitis, with patients presenting with cough and dyspnea within 6 months of starting treatment, or as a late fibrosis with gradual worsening dyspnea and a nonproductive cough (Table 1).^2^

**Table 1:** Teaching points related to the case

1. Cyclophosphamide, a commonly used alkylating agent for the treatment of ANCA-associated vasculitis, can cause direct lung toxicity
2. Lung damage can manifest as early-onset pneumonitis (likely reversible) or late-onset lung fibrosis (usually irreversible)
3. When considering patients undergoing hemodialysis who present with symptoms and signs of cyclophosphamide-associated lung injury, it is important to first exclude pulmonary infection and edema
4. In patients with a pneumonitis-type picture, as presented here, withdrawal of cyclophosphamide can lead to resolution of symptoms and radiographic evidence of disease

ANCA, antineutrophil cytoplasmic antibody.

**Figure 2:**
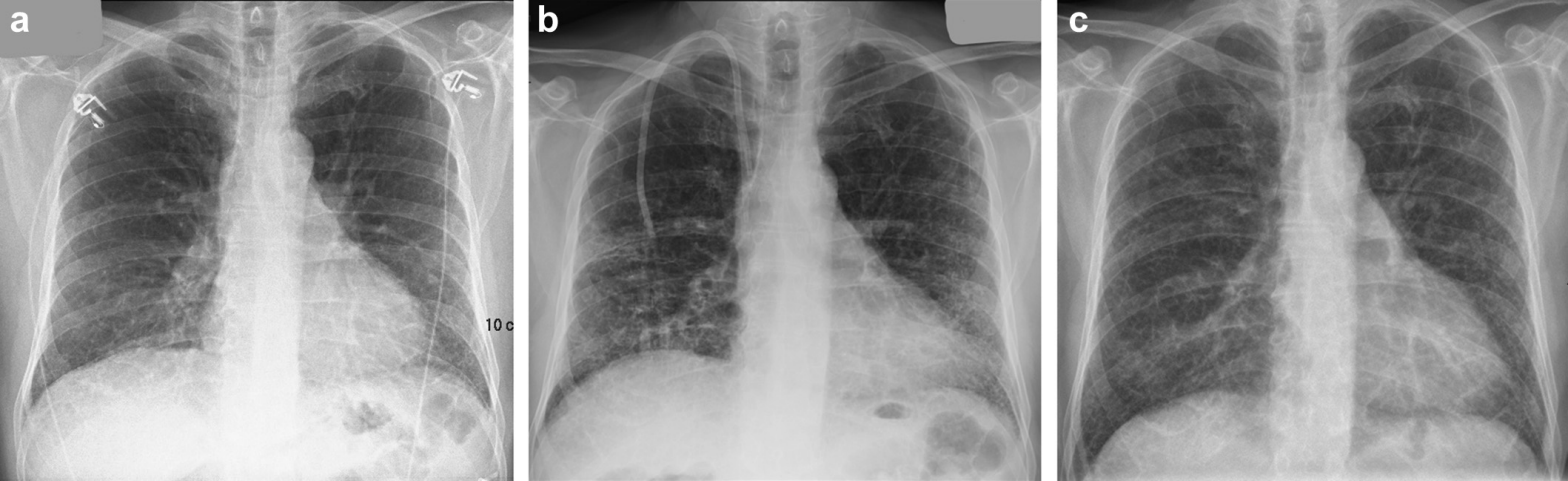
(a) X-ray taken before cyclophosphamide treatment. (b) X-ray taken during treatment. (c) X-ray taken 2 months after cyclophosphamide was discontinued. The pretreatment X-ray was reported as showing a largely peripheral and basal pattern of fibrosis, which then improved significantly following drug withdrawal.

The diagnosis of cyclophosphamide-related lung injury is made clinically on the basis of symptoms, history of cyclophosphamide use, compatible findings on chest imaging studies, and the absence of an alternative diagnosis. Early drug toxicity may respond to discontinuation of cyclophosphamide alongside glucocorticoids, whereas late toxicity is usually untreatable. It is unclear whether a higher total dose of cyclophosphamide is a risk factor for lung toxicity, as affected patients have received doses ranging from 150 mg to 81 g.^2^, ^3^ As demonstrated here, HRCT may be a better imaging modality than plain chest X-ray for detecting these changes (serial chest X-rays for the patient described here are shown in Figure 2).

The importance of recognizing the pulmonary toxicity associated with cyclophosphamide is underscored by the fact that the prevalence rates of ANCA-associated vasculitis, for which cyclophosphamide is the main therapeutic agent, are increasing, as are the global rates of cancer, another indication for treatment with this agent.^4^, ^5^ Furthermore, the sales of cyclophosphamide in the United States alone for the year ending September 2014 amounted to ∼$420 million according to IMS Health. There have been few data published over the past 20 years detailing the important pulmonary toxicity associated with the drug described here. When considering cyclophosphamide-related lung injury in those patients receiving maintenance hemodialysis, fluid overload should first be excluded.

## Patient Perspective

### Diagnosis and Initial Treatment

*“I remember feeling constantly tired and exhausted… I could hardly walk the length of my front room without feeling breathless. The treatment worked really well for my breathing… But of course, what was really beginning to worry me at that time was being started on dialysis…”*

### Re-admission

*“Life was much more difficult after I made it home the first time… The diagnosis began to sink in, I still had to travel to the hospital three times a week for dialysis, and I came to rely on those around me for a lot of things that I could do myself before, like shopping and cooking…*

*After a while, though, I noticed I was becoming more short of breath and struggling whenever I left the house… It got to the stage where I could hardly speak without feeling short of breath. That’s when I decided enough was enough and I needed help again. Things have taken a lot longer to get better this time, but it still feels like things are going in the right direction…”*

## Disclosure

All the authors declared no competing interests.
